# Trade-offs between xylem hydraulic efficiency and mechanical strength in Chinese evergreen and deciduous savanna species

**DOI:** 10.1093/treephys/tpac017

**Published:** 2022-02-14

**Authors:** Shu-Bin Zhang, Guo-Jing Wen, Ya-Ya Qu, Lin-Yi Yang, Yu Song

**Affiliations:** CAS Key Laboratory of Tropical Forest Ecology, Xishuangbanna Tropical Botanical Garden, Chinese Academy of Sciences, Mengla, Yunnan 666303, China; Yuanjiang Savanna Ecosystem Research Station, Xishuangbanna Tropical Botanical Garden, Chinese Academy of Sciences, Yuanjiang, Yunnan 653300, China; CAS Key Laboratory of Tropical Forest Ecology, Xishuangbanna Tropical Botanical Garden, Chinese Academy of Sciences, Mengla, Yunnan 666303, China; Yuanjiang Savanna Ecosystem Research Station, Xishuangbanna Tropical Botanical Garden, Chinese Academy of Sciences, Yuanjiang, Yunnan 653300, China; School of Forestry, Southwest Forestry University, No. 300, Bailongshi, Panlong District, Kunming, Yunnan 650224, China; School of Forestry, Southwest Forestry University, No. 300, Bailongshi, Panlong District, Kunming, Yunnan 650224, China; Center for Integrative Conservation, Xishuangbanna Tropical Botanical Garden, Chinese Academy of Sciences, Mengla, Yunnan 666303, China

**Keywords:** Chinese savanna, fiber content, hydraulic conductivity, modulus of rupture, trade-off, vessel anatomy, wood density

## Abstract

Evergreen and deciduous species coexist in tropical dry forests and savannas, but differ in physiological mechanisms and life-history strategies. Hydraulic conductivity and mechanical support are two major functions of the xylems of woody plant species related to plant growth and survival. In this study, we measured sapwood-specific hydraulic conductivity (K_s_), leaf-specific hydraulic conductivity (K_L_), modulus of rupture (MOR) and elasticity (MOE), xylem anatomical traits and fiber contents in the xylems of 20 woody species with contrasting leaf phenology (evergreen vs deciduous) in a Chinese savanna. Our results showed that deciduous species had significantly higher K_s_ and K_L_ but lower MOR and MOE than evergreen species. Evergreen species experienced more negative seasonal minimum water potential (P_min_) than deciduous species during the dry season. Furthermore, we found trade-offs between xylem hydraulic efficiency and mechanical strength across species and within the evergreen and deciduous groups, and these trade-offs were modulated by structural and chemical traits. Both K_s_ and K_L_ were significantly related to hydraulic weighted vessel diameter (D_h_) across all species and within the deciduous group. Both MOR and MOE were significantly related to wood density, neutral detergent fiber and acid detergent fiber across species and within evergreen and deciduous groups. Our findings demonstrated that Chinese evergreen and deciduous savanna species diverged in xylem hydraulic and mechanical functions, reflecting conservative and acquisitive life-history strategies for evergreen and deciduous species, respectively. This study provides new information with which to understand the hydraulic and biomechanical properties and ecological strategies of savanna species in long-term dry-hot environments.

## Introduction

The xylem of woody plants is of great importance in long-distance water transport, mechanical support, and the storage of water, carbohydrates and nutrients (Wagner et al. 1998, King et al. 2006, Pratt and Jacobsen 2017, van der Sande et al. 2019, Pratt et al. 2021*a*). The advantages of efficient water transport in the xylem can maintain leaf water potential at a relatively high value, keep the stomata open and maintain photosynthetic gas exchange (Brodribb and Feild 2000, Pratt and Jacobsen 2017, Liu et al. 2019). Previous studies have shown that xylem hydraulic conductivity is correlated with leaf photosynthetic capacity (Brodribb and Feild 2000, Santiago et al. 2004), plant growth rate (Fan et al. 2012, Hoeber et al. 2014, Qi et al. 2021), productivity (Zhao et al. 2021) and even species distribution (Markesteijn et al. 2011, Martin et al. 2013). In addition, xylem mechanical strength protects plants from abiotic and biotic damage, which affects plant survival, growth and reproductive performance (Wagner et al. 1998, King et al. 2006, Read and Stokes 2006, Onoda et al. 2010, Pratt et al. 2021*a*). A plant needs to maintain a certain level of water transport to allow leaf transpiration and photosynthesis, as well as adequate mechanical support to resist breaking forces from physical disturbance and herbivores (Wagner et al. 1998, Onoda et al. 2010, Lachenbruch and McCulloh 2014). Both xylem hydraulic efficiency and mechanical strength are closely linked to the acquisition and use of water and nutrient resources, the competitive ability and the life-history strategies of the plants (Choat et al. 2005, Sterck et al. 2006, Markesteijn et al. 2011, Zhang et al. 2013, Pratt et al. 2021*a*). It is important for us to understand whether hydraulic efficiency and mechanical strength in the xylems of woody plants are related to each other and how anatomical traits shape the covariation of hydraulic and mechanical performance (Lachenbruch and McCulloh 2014, Pratt and Jacobsen 2017).

Sapwood-specific hydraulic conductivity (K_s_) can quantify the water transport ability in the xylem, whereas the leaf-specific hydraulic conductivity (K_L_) indicates the hydraulic efficiency of the xylem to supply water to the leaves (Zimmermann 1978, Sperry and Tyree 1988). The anatomical traits of conductive tissues (vessel in angiosperm or tracheid in gymnosperm) affect hydraulic efficiency in the xylem (Tyree et al. 1994). In general, wider and longer vessels can support higher hydraulic efficiency than narrower and shorter vessels (Hacke et al. 2006, Lens et al. 2011). Moreover, the modulus of elasticity (MOE) and modulus of rupture (MOR) indicate the bending stiffness and resistance to breaking in the branched xylem (Gere and Timoshenko 1999, Pratt and Jacobsen 2017). Numerous studies have suggested that both MOR and MOE are well correlated with wood density (WD) across species (King et al. 2006, Pratt et al. 2007, Onoda et al. 2010, Santini et al. 2013, Xiao et al. 2021, Pratt et al. 2021*a*). In gymnosperm species, both water transport and mechanical support are provided by tracheid-based systems (Pittermann et al. 2006). In angiosperm species, fibers are involved in mechanical support, and xylem mechanical stiffness and resistance are related to fiber traits, such as the proportion of fibers in xylem cross section, fiber lumen diameter (FLD), fiber wall thickness (FWT), fiber arrangement and cellulose microfibril angle (Lachenbruch and McCulloh 2014, Pratt and Jacobsen 2017, L. Zhang et al. 2019, Pratt et al. 2021*a*). Furthermore, the fibers in leaf tissues can support the skeleton in cell wall composition (Kitajima et al. 2012), and lignin can increase the bending resistance (Alvarez-Clare and Kitajima 2007). Leaf fracture toughness was related to cellulose content per dry mass (DM) across species and within plants (Kitajima et al. 2016). To our knowledge, however, there have been no tests for the relationships between fiber content and mechanical strength in xylems.

The division of labor between vessels for water transport and fibers for mechanical support suggests an interdependency between hydraulic efficiency and mechanical strength in the xylem of angiosperm species (Pratt and Jacobsen 2017). Some studies have shown a trade-off between water transport efficiency and mechanical strength (Wagner et al. 1998, Fan et al. 2017, Xiao et al. 2021), however, others have shown no relationship at all (Jacobsen et al. 2007*a*, L. Zhang et al. 2013). Furthermore, previous studies have also found trade-offs between xylem mechanical traits and starch storage, and between storage capacity and embolism resistance under drought, and these trade-offs were mitigated by the structural and functional basis of xylems (Chen et al. 2020, Pratt et al. 2021*b*). More studies are needed to make a comprehensive conclusion regarding the potential trade-offs or associations between xylem hydraulic efficiency and mechanical strength. Environmental conditions may put selective pressure on the trade-offs or associations among xylem xylems (Fu et al. 2012, Sanchez-Martinez et al. 2020, Pratt et al. 2021*a*). In seasonal environments, water limitation strongly influences xylem functions (Pratt et al. 2021*b*), and the minimum water potential experienced by plants during drought stress (P_min_) indicates the drought exposure in the tissues, which is determined by multiple traits such as plant drought tolerance, hydraulic safety and leaf phenology (Bartlett et al. 2016, Zhang et al. 2017, Sanchez-Martinez et al. 2020, Pratt et al. 2021*a*). Pratt et al. (2021*a*) also revealed that P_min_, as a hub trait, was linked to cellular trade-offs of fiber, vessel and parenchyma, which drove the functional trade-offs among xylem strength, transport and storage.

Evergreen and deciduous woody species coexist in seasonally dry tropical forests and savannas, because of the strong selective pressure of seasonal drought (Quigley and Platt 2003, Choat et al. 2005, Ishida et al. 2010, Fu et al. 2012, Zhang et al. 2017). Several studies have shown that co-occurring evergreen and deciduous species exhibit clear distinctions in stem hydraulic efficiency (Chen et al. 2009, Fan et al. 2011, Fu et al. 2012); however, others have failed to find this divergence (Ishida et al. 2010). The elucidation of this issue is further complicated by the lack of a direct test to compare the potential differences in biomechanical traits between evergreen and deciduous species.

Savanna ecosystems are mainly distributed in tropical and subtropical regions, covering appropriately 20% of the land surface and accounting for 30% of the primary production of terrestrial vegetation (Solbrig et al. 1996, Grace et al. 2006). Valley-type savanna occurs widely in the deep, hot and dry valleys of several large rivers among the high mountains, in Southwest China (Jin and Ou 2000, Zhu et al. 2020). Because of strong seasonality in the precipitation of savanna ecosystem, the environmental pressure drives the differentiation in leaf phenology, and evergreen and deciduous woody species coexist in this Chinese savanna (Zhang et al. 2007, 2017). Our previous studies have shown that evergreen and deciduous tree species exhibit divergent strategies of drought tolerance and hydraulic safety under prolonged seasonal drought (Zhang et al. 2017, SB Zhang et al. 2019). However, the ecological strategies in hydraulic efficiency and mechanical strength have not been well characterized for Chinese savanna woody species with contrasting leaf phenology.

In this study, we measured 14 xylem functional traits related to hydraulic efficiency and biomechanical strength, as well as P_min_ in woody species (7 evergreen species vs 13 deciduous species) in a savanna ecosystem, Southwest China. We evaluated the potential differences in hydraulics and biomechanics between evergreen and deciduous savanna species, and elucidated the possible trade-offs and associations between hydraulic and biomechanical functions and underlying anatomical traits. Given phylogenetic conservatism, traits at the species level are influenced by evolutionary history, and closely related species are expected to have similar trait values (Blomberg et al. 2003, Losos 2008). Evolutionary correlations among hydraulic, biomechanical, anatomical traits and fiber contents were also analyzed using phylogenetically independent contrasts (PICs; Paradis and Schliep 2019). We addressed the following questions: (i) do evergreen and deciduous species differ in terms of hydraulic efficiency and mechanical strength; (ii) is there an evolutionary trade-off between xylem hydraulic efficiency and mechanical strength across species and within evergreen and deciduous groups; and (iii) how is the hydraulic and mechanical trade-off, if any, determined by the structural traits?

## Materials and methods

### Study sites

This study was conducted at Yuanjiang Savanna Ecosystem Research Station (YSERS, 23°27′N, 102°10′E and 481-m elevation above sea level), Xishuangbanna Tropical Botanical Garden, Chinese Academy of Sciences, located in Yuanjiang County, Yunnan Province, Southwest China. The study site hosts a valley-type savanna (Jin and Ou 2000). The climate is characterized by two distinct seasons: rainy season (May–October) and dry season (November–April). According to the meteorological record of YSERS observed from 2012 and 2020, the mean annual temperature was 25.0 °C with a mean monthly temperature ranging from 17.7 °C (January) to 30.3 °C (June). The total annual precipitation was 675.8 mm, concentrated from June to September (see Figure S1 available as Supplementary data at *Tree Physiology* Online). Due to rock outcrops (60–70%), water leakage and shallow soil, this valley-type savanna lacks groundwater reserves, and water stress is a dominant selective pressure (Jin and Ou 2000). The soil is ferralic cambisol with pH of 7.2 ± 0.1. The concentrations of nitrogen, phosphorus, potassium and sulfur in the topsoil (0–20 cm) were 0.285 ± 0.022%, 0.086 ± 0.006%, 1.673 ± 0.012% and 0.031 ± 0.002%, respectively.

### Plant materials

Based on the species composition of the 1 ha (100 m × 100 m) long-term monitoring Chinese savanna plot, we selected 13 deciduous and 7 evergreen plant species in this study, including 12 families and 20 genera (Table 1). These deciduous species shed their leaves during the dry season. These 20 dominant species account for >70% of the biomass in the woody species of this valley-type savanna in Southwest China. The hydraulic and mechanical traits of the plants may be affected by the availability of light over the crown (Raimondo et al. 2009, Markesteijn et al. 2011). Therefore, all the sampled trees were completely exposed to overhead light to minimize the effects of shade. Five sun-exposed terminal branches were sampled from three to five adult individuals of each species, during the rainy season (July–August) in 2014. Sampled branches with healthy leaves were wrapped in moist towels and transported in a sampling box to the YSERS laboratory for further measurements. In each sample and measurement cycle, we only collected the branches of two species and kept them at 4 °C before hydraulic measurements. We measured hydraulic traits within 2 days after sampling the branches in the field.

**Table 1:** Characteristics of 20 evergreen and deciduous species included in the present study.

Species	Code	Family	Phenology	Growth form	Canopy height
*Haldina cordifolia* (Roxb.) Ridsd.	Hc	Rubiaceae	Deciduous	Tree	5–7 m
*Cipadessa cinerascens* (Pellegr.) Hand	Cc	Meliaceae	Deciduous	Shrub	3 m
*Polyalthia cerasoides* (Roxb.) Benth. & Hook.f. exBedd.	Pc	Annonaceae	Deciduous	Tree	3–5 m
*Vitex negundo* L.	Vn	Verbenaceae	Deciduous	Shrub	4 m
*Trigonostemon tuberculatum* F.Du et J.He sp. nov.	Tt	Euphorbiaceae	Deciduous	Shrub	3 m
*Bauhinia brachycarpa* Benth.	Bb	Leguminosae	Deciduous	Shrub	3 m
*Lannea coromandelica* (Houtt.) Merr.	Lc	Anacardiaceae	Deciduous	Tree	5–8 m
*Terminalia franchetii* Gagnep.	Tf	Combretaceae	Deciduous	Tree	5 m
*Bridelia stipularis* (L.) Blume	Bs	Euphorbiaceae	Deciduous	Liana	5 m
*Strophioblachia fimbricalyx* Boerl.	Sf	Euphorbiaceae	Deciduous	Shrub	3 m
*Terminthia paniculata* (Wall. ex G. Don) C. Y. Wu et T. L. Ming	Tp	Anacardiaceae	Deciduous	Shrub	5 m
*Woodfordia fruticosa* (L.) Kurz	Wf	Lythraceae	Deciduous	Shrub	2–4 m
*Campylotropis delavayi* (Franch.) Schindl.	Cd	Leguminosae	Deciduous	Tree	4 m
*Tarenna depauperata* Hutch.	Td	Rubiaceae	Evergreen	Shrub	3 m
*Pistacia weinmanniifolia* J. Poiss. exFranch.	Pw	Anacardiaceae	Evergreen	Tree	5–7 m
*Diospyros yunnanensis* Rehder & E. H. Wilson	Dy	Ebenaceae	Evergreen	Tree	4–5 m
*Olea europaea* L.	Oe	Oleaceae	Evergreen	Tree	4–6 m
*Psidium guajava* L.	Pg	Myrtaceae	Evergreen	Tree	6–8 m
*Carissa spinarum* L.	Cs	Apocynaceae	Evergreen	Tree	3 m
*Burretiodendron kydiifolium* Hsu et Zhuge	Bk	Malvaceae	Evergreen	Tree	6 m

### Hydraulic traits

The maximum vessel length (MVL) was determined using the air injection method (Greenidge 1952). To avoid any effect of open vessels on our measurements of hydraulic conductivity, we sampled branches longer than the MVL. However, previous studies have also suggested that sample length has very little effect on the measured hydraulic conductivity in cut stems (Sperry et al. 2005, Jacobsen et al. 2015). These branches were recut under distilled water and trimmed using a razor blade. Potassium chloride (0.1 mol l^−1^ KCl) solution was used to flush the stem segments for at least 20 min to remove the native emboli present within the xylem vessels. The pressure of the KCl solution was set at 100 kPa. We measured the water flow (*F*, kg s^−1^) using the drop in pressure to generate a gravity-induced hydrostatic pressure (7.3 kPa), whereas the stem segments were connected to a hydraulic conductivity apparatus (Sperry and Tyree 1988). We determined hydraulic conductivity (K_h_, kg m MPa^−1^ s^−1^) as the flow rate divided by the pressure gradient. Subsequently, the cross-sectional area of the distal end of the samples (A_s_, mm^2^) was determined as the average value of the two ends of the stem segments measured for hydraulic conductivity. Sapwood-specific hydraulic conductivity (K_s_, kg m^−1^ MPa^−1^ s^−1^) was calculated by K_h_ divided by A_s_.

The total leaf area (A_L_) distal to the base of the cut stem was scanned using a Li-3000A leaf area meter (Li-cor, Lincoln, NE, USA). We calculated leaf-specific hydraulic conductivity (K_L_, kg m^−1^ MPa^−1^ s^−1^) by K_h_ divided by A_L_. The Huber value (HV, mm^2^ m^−2^) is the ratio of sapwood area to leaf area, which indicates the relative allocation to sapwood area and leaf area and is central to the plant water balance of the branches and leaves of the canopy (Maurizio et al. 2019).

### Young’s modulus

Following the determination of hydraulic traits, the same stem segments that had been used for hydraulic measurements were used to measure the MOE and MOR with a three-point bending method using a universal testing machine (Model 3343, Instron, Illinois Tool Works Inc., Norwood, MA, USA). The diameter-to-length ratio was set to 1:20. The vertical force was applied at a speed of 20 mm min^−1^. The bark, including the outer bark, phloem and cambium layers, was carefully removed before mechanical measurements. For each sample without bark, we measured the diameters at the midpoint and at both ends with a caliper (0.01 mm) and calculated its radius (*R*). The MOR was estimated by:

(1) \begin{equation*} \mathrm{MOR}={F}_{\mathrm{max}}\times L\times R/4I \end{equation*}

where *F*_max_ is the maximum force (N) when the debark stems are broken, *L* is the span length of the supports (m), *R* is the mean radius of debark stems (m) and *I* is the second moment of area (m^4^), which was calculated as follows (Gere and Timoshenko 1999):

(2) \begin{equation*} I=\pi{R}^4/4 \end{equation*}

The MOE was calculated from the linear region of the relationships between the load force (*F*, N) and its corresponding deflection (*δ*, mm; Gere and Timoshenko 1999):

(3) \begin{equation*} \mathrm{MOE}=F{L}^3/48 I\delta \end{equation*}

### Wood density and anatomy

After measuring the hydraulic and mechanical traits, one 5-cm segment from each debarked stem was used to measure the WD (g cm^−3^). The volume of the fresh wood (*V*_wood_) was measured using the water displacement method. The DM was weighed after drying in an oven at 70 °C for 72 h. Wood density was calculated as follows:

(4) \begin{equation*} \mathrm{WD}=\mathrm{DM}/{V}_{\mathrm{wood}} \end{equation*}

Five segments per species were sampled for anatomical traits. Segments 3 cm in length were sampled from the debarked stems used for the mechanical and hydraulic traits. Transverse sections, 20-μm thick, were made with a microtome and stained with a mixture of safranin and Alcian blue. Digital photographs of the xylem transverse sections were recorded using a DM2500 light microscope (Leica Inc., Bensheim, Germany) connected to a computer. Three digital images per stem were taken to analyze the anatomical traits using ImageJ software (http://rsbweb.nih.gov/ij/). At least 100 vessel and fiber cells per species were used to determine the vessel and fiber traits.

Vessel density (VD, no. of vessels mm^−2^) was calculated as the number of vessels per xylem area. Moreover, we measured the diameter of each vessel (D_v_) and calculated the hydraulic weighted vessel diameter (D_h_, μm) as follows:

(5) \begin{equation*} {\mathrm{D}}_{\mathrm{h}}={\left(\sum{{\mathrm{D}}_{\mathrm{v}}}^4/\mathrm{N}\right)}^{1/4} \end{equation*}

where *N* is the number of vessels (Tyree and Zimmermann 2002).

In addition, we measured the FLD (μm) and FWT (μm) from the digital photographs. We measured the inter-FWT from two adjacent fiber cells; this was halved to calculate the FWT (Antonio et al. 2017).

### Fiber content

Fresh wood without bark was sampled to measure the fiber content. After drying at 70 °C for at least 96 h, the wood samples were ground to a fine powder with a crusher and passed through a 20-mesh sieve. Neutral detergent fiber (NDF) indicates the total content of hemicellulose, cellulose and lignin, and acid detergent fiber (ADF) indicates the total contents of cellulose and lignin (Van Soest 1994, Onoda et al. 2011). We measured the proportions of NDF and ADF on an ash-free mass basis using the Van Soest method (Van Soest 1994). Then, NDF (g cm^−3^) and ADF (g cm^−3^) were recalculated by the product of WD and their proportions of NDF and ADF in wood DM. Thus, NDF and ADF were expressed per unit volume.

### Seasonal minimum water potentials

The seasonal minimum water potential (P_min_) was measured between 12:00 h and 14:00 h on sunny days during the dry season from November 2013 to April 2014. We wrapped the leaf-bearing branches for evergreen species and leafless terminal twigs for deciduous species with plastic bags and aluminum foil in the evening before the measurement days. We assumed that the leaf and branch water potentials were equilibrated. The midday water potentials of bagged leaves were measured using a pressure chamber (PMS). Because of leaf-shedding in deciduous species, we used leafless terminal twigs to determine stem P_min_ during the dry season. Our recent study in this Chinese savanna found that the were no significant differences when determining stem water potentials using leaf and leafless terminal twigs after fully equilibrium (Chen et al. 2021). Seasonal minimum water potential (P_min_) was the lowest midday water potential under natural conditions during dry periods.

### Phylogeny tree

We present phylogenetic analyses of rbcL sequences (see Table S3 available as Supplementary data at *Tree Physiology* Online) from 20 species from GenBank (https://www.ncbi.nlm.nih.gov/). The alignments were carried out in Clustal X (Aiyar 2000). Maximum likelihood analysis was carried out using IQ-TREE v.2.1.1 software (Minh et al. 2020). The DNA substitution models was chosen as ‘GTR + F + R3’. One thousand bootstrap replicates were performed for each analysis to obtain confidence support. Finally, a phylogeny tree at the species level was constructed for all 20 species in this study (Figure S2 available as Supplementary data at *Tree Physiology* Online).

### Data analysis

All statistical analyses were conducted using Rstudio with R 3.6.3 (R Development Core Team 2017). We compiled a dataset with species mean functional trait values. Species with greater absolute values of P_min_ (−P_min_) would experience more severe seasonal water stress. The values of P_min_ were converted from negative to positive (−P_min_) in principal component analysis (PCA) and correlation analyses. All traits were tested for normality using the Shapiro–Wilk test (Patrick 1982), and logarithm-transformed as necessary to improve normality and homoscedasticity before statistical analyses (Table S1 available as Supplementary data at *Tree Physiology* Online). The differences in traits between the deciduous and evergreen groups were tested using one-way analysis of variance (ANOVA). Principal component analysis was performed to evaluate the multiple relationships among functional traits and species, using the ‘FactoMinerR’ package (Lê et al. 2008).

Pearson’s correlation analyses were conducted to determine the bivariate relationships among traits using the ‘corr.test’ function of the ‘psych’ package. The phylogenetic signal was tested for every trait using Blomberg’s *K* statistic (Blomberg et al. 2003). A value of *K* > 1 indicated a strong phylogenetic signal of traits, whereas a value of *K* close to 0 indicated no phylogeny signal of traits (Blomberg et al. 2003). Furthermore, we assessed whether these correlations were the result of repeated evolutionary divergences using PICs combining the ‘APE’ package (Paradis and Schliep 2019), and ‘Picante’ package in R (Webb et al. 2010).

Standardized major axis (SMA) regression was used to test the bivariate relationships between hydraulic efficiency traits (K_s_ and K_L_) and biomechanical traits (MOE and MOR). The relationships of hydraulic and biomechanical traits with structural and anatomical traits were also analyzed using the ‘smatr’ package (Warton and Weber 2002). We compared the differences in the slopes and intercepts between the deciduous and evergreen groups.

## Results

Deciduous species had higher K_s_, K_L_ and D_h_, but lower MOR, MOE, VD, VWT and FLW, than evergreen species (*P* < 0.05, Table 2). In addition, evergreen species had a more negative P_min_ (−2.94 ± 0.17 MPa) compared with the deciduous group (−4.03 ± 0.27 MPa; *P* < 0.05, Table 2). Evergreen and deciduous species did not, however, significantly differ in HV, MVL and FLD (*P* > 0.05, Table 2). For all traits, Blomberg’s *K*-values were <1, and the corresponding *P*-values were higher than 0.05 (see Table S4 available as Supplementary data at *Tree Physiology* Online), indicating no phylogenetic signal.

**Table 2:** The lists of functional traits, their abbreviations and units in this study, and the comparisons of hydraulic, mechanical and anatomical traits between evergreen and deciduous species in a savanna ecosystem, Southwest China. Significance levels: ^*^, 0.01 < *P* < 0.05; ^**^, 0.001 < *P* < 0.01; ^***^, *P* < 0.001 and *ns*, non-significant.

Functional traits	Abbreviation	Unit	Deciduous	Evergreen	Significance
Sapwood-specific hydraulic conductivity	K_s_	kg m^−1^ s^−1^ MPa^−1^	3.59 ± 0.46	1.23 ± 0.21	**
Leaf-specific hydraulic conductivity	K_L_	10^−4^ kg m^−1^ s^−1^ MPa^−1^	5.51 ± 0.89	1.64 ± 0.43	***
Huber value	HV	mm^2^ m^−2^	1.53 ± 0.16	1.30 ± 0.16	*ns*
Maximum vessel length	MVL	cm	46.1 ± 4.0	52.3 ± 4.4	*ns*
Modulus of rupture	MOR	MPa	73.8 ± 4.4	110.9 ± 11.5	**
Modulus of elasticity	MOE	MPa	5434.1 ± 400.3	8289.3 ± 830.5	**
Mean hydraulically weighted diameter	D_h_	μm	52.0 ± 3.2	31.2 ± 1.9	***
Vessel density	VD	no. mm^−2^	79.5 ± 10.1	171.3 ± 27.1	**
Vessel wall thickness	VWT	μm	5.5 ± 0.4	6.8 ± 0.3	*
Fiber wall thickness	FWT	μm	4.9 ± 0.3	8.7 ± 0.4	***
Fiber lumen diameter	FLD	μm	13.3 ± 0.8	13.0 ± 0.9	*ns*
Wood density	WD	g cm^−3^	0.54 ± 0.02	0.67 ± 0.04	**
Neutral detergent fiber	NDF	g cm^−3^	0.45 ± 0.02	0.610 ± 0.02	**
Acid detergent fiber	ADF	g cm^−3^	0.42 ± 0.04	0.569 ± 0.04	*
Seasonal minimum water potential	P_min_	MPa	−2.94 ± 0.17	−4.03 ± 0.27	**

**Figure 1.**
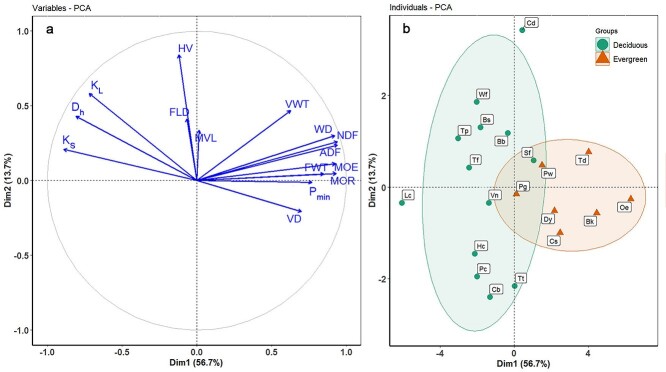
Factor loadings (a) and species scores (b) PCA for 15 functional traits and 20 species in a savanna ecosystem, Southwest China. Species codes and trait abbreviations are given in Tables 1 and 2, respectively.

Principal component analysis results based on the 15 traits of 20 savanna woody species showed that the first axis and second axis accounted for 56.7 and 13.7% of the total variance, respectively (Figure 1). The first axis was positively correlated with traits representative of high mechanical strength (i.e., MOR, MOE, WD, NDF and ADF) and strong drought exposure (−P_min_). The traits related to high hydraulic efficiency (i.e., K_s_, K_L_ and D_h_) clustered negatively on the first axis. The second axis was loaded by HV, MVL and FLD (Figure 1a). In addition, evergreen and deciduous woody species were well separated along the first PCA axis. Deciduous species mainly tended to be positioned on the negative side of the first axis with high hydraulic efficiency, whereas evergreen species tended to be positioned on the positive side of the first axis with high mechanical strength and strong drought exposure (−P_min_; Figure 1b). However, there was some overlap between a deciduous shrub species (*Strophioblachia fimbricalyx*) and two evergreen tree species (*Pistacia weinmanniifolia* and *Psidium guajava*; Figure 1b).

**Figure 2.**
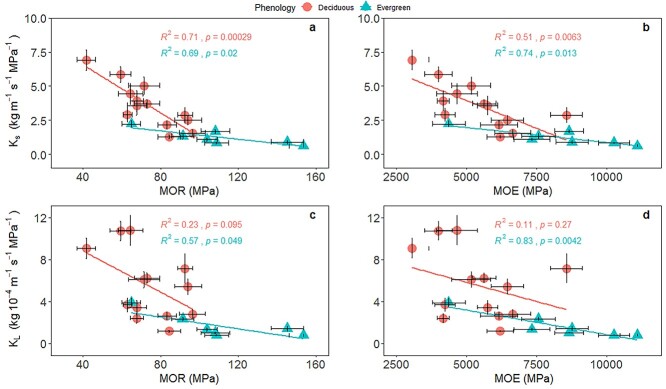
Relationships between hydraulic efficiency and biomechanical traits in the xylems of evergreen and deciduous species in a savanna ecosystem, Southwest China. Trait abbreviations are shown in Table 2. Standardized major axis (SMA) regression was fitted for the evergreen and deciduous groups, respectively. See Table S2 available as Supplementary data at *Tree Physiology* Online for SMA regression results.

Both MOR and MOE were negatively related to K_s_ either across the 20 studied species, or separately within evergreen and deciduous species (Figure 2, Tables S2 and S5 available as Supplementary data at *Tree Physiology* Online). For the regressions of K_s_ and mechanical traits (MOR and MOE), the SMA slopes were significant, but the intercepts were no significant between the deciduous and evergreen groups (Table S2 available as Supplementary data at *Tree Physiology* Online). There was a significant correlation between MOR and K_L_, and between MOE and K_L_ across all species (Figure 2 and Table S2 available as Supplementary data at *Tree Physiology* Online). Both MOE and MOR were also negatively correlated with K_s_ and K_L_ when analyzed using PICs (*P* < 0.001, Table S5 available as Supplementary data at *Tree Physiology* Online). Species with higher xylem mechanical strength tend to have lower hydraulic efficiency. We found trade-offs between xylem hydraulic efficiency and mechanical strength irrespective of the effects of phylogeny.

**Figure 3.**
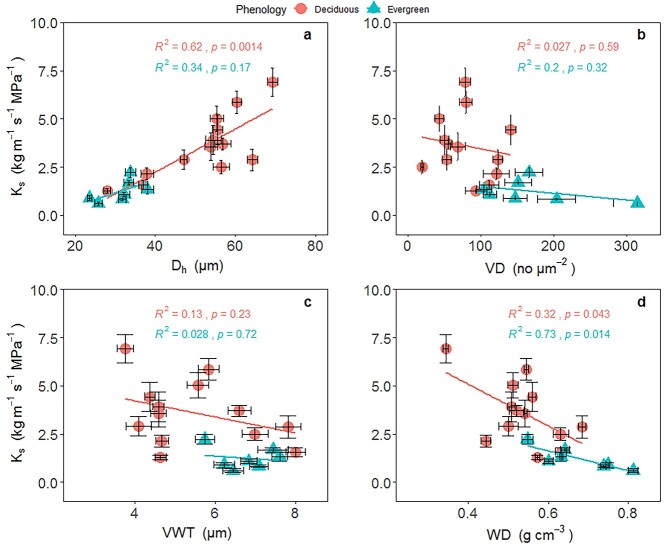
Relationships between sapwood-specific hydraulic conductivity (K_s_) and anatomical traits within evergreen and deciduous groups in a savanna ecosystem, Southwest China. Trait abbreviations are shown in Table 2. Standardized major axis regression was fitted for the evergreen and deciduous groups, respectively. See Table S2 available as Supplementary data at *Tree Physiology* Online for SMA regression results.

**Figure 4.**
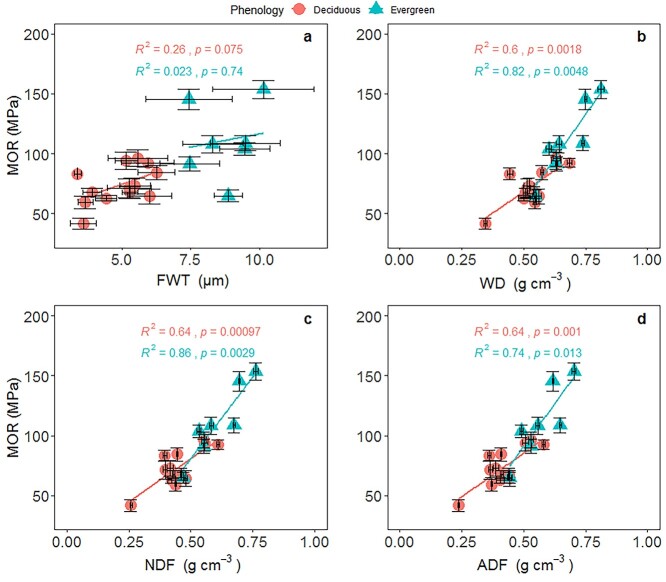
Relationships between MOR and structural traits within evergreen and deciduous groups in a savanna ecosystem, Southwest China. Trait abbreviations are shown in Table 2. Standardized major axis (SMA) regression was fitted for the evergreen and deciduous groups, respectively. See Table S2 available as Supplementary data at *Tree Physiology* Online for SMA regression results.

The SMA regressions across all 20 species showed that K_s_ was significantly and positively associated with D_h_, but negatively correlated with VD, WD and VWT (*P* < 0.05, Figure 3, Table S2 available as Supplementary data at *Tree Physiology* Online). Both MOR and MOE were significantly correlated with FWT (*P* < 0.05), but were not significantly related to FLD across species (*P* > 0.05, see Table S5 available as Supplementary data at *Tree Physiology* Online). MOR was positively related to WD, NDF and ADF across all 20 species or within evergreen and deciduous species, respectively (*P* < 0.05, Figure 4). Furthermore, the SMA slopes were marginally different between the deciduous and evergreen groups for the regressions of mechanical traits (MOR) with WD and fiber contents (NDF and ADF; see Table S2 available as Supplementary data at *Tree Physiology* Online).

## Discussion

In this study, we compared the hydraulic efficiency and mechanical strength of evergreen and deciduous woody species in a Chinese savanna ecosystem, and tested the relationships between hydraulic and mechanical functions and underlying structural and anatomical traits. Our results revealed a distinct divergence in hydraulic and mechanical traits between the evergreen and deciduous groups. Specifically, deciduous species had a higher xylem hydraulic efficiency, whereas evergreen species had higher mechanical strength. In addition, xylem anatomical traits and fiber contents could explain the hydraulic and mechanical functioning of the Chinese savanna evergreen and deciduous species.

In this study, none of the hydraulic, mechanical, anatomical or chemical traits showed phylogenetic signals. Moreover, the bivariate correlations between traits using PICs were similar to those with raw values of functional traits, suggesting that pairwise relationships between traits were phylogenetically independent in Chinese savanna woody species. Our result was consistent with previous studies in chaparral shrubs of southern California (Pratt et al. 2021*a*), and tree and liana species of tropical seasonal rainforest in Southwest China (L. Zhang et al. 2019). These results suggested that phylogeny cannot explain the associations among the plant xylem functions well. Here, we discuss the differences between evergreen and deciduous species, and the trade-offs between hydraulic efficiency and mechanical strength and the underlying anatomical and chemical traits irrespective of phylogeny.

### Evergreen and deciduous species differ in hydraulic efficiency and mechanical strength

Our study was conducted in a semiarid savanna ecosystem where there was a seasonal drought from November to April (Figure S1 available as Supplementary data at *Tree Physiology* Online). This strong environmental pressure drove ecological differentiation in the leaf phenology of savanna species (Zhang et al. 2007, 2017). Co-occurring evergreen and deciduous species differ in terms of stem hydraulic conductivity and water use strategies in seasonally tropical dry forests and savannas (Eamus 1999, Chen et al. 2009, Ishida et al. 2010, Markesteijn et al. 2011, Fu et al. 2012, Zhang et al. 2017, S.B. Zhang et al. 2019). Previous studies have shown that P_min_ acts on plant functional trade-offs under drought stress (Jacobsen et al. 2007*b*, Pratt et al. 2007, Bartlett et al. 2016, Pratt et al. 2021*b*). In the present study, deciduous woody species exhibited higher K_s_, K_L_ and D_h_, whereas evergreen woody species had higher VD and VWT values. Moreover, the xylems experienced more negative water potential (P_min_) in evergreen species than in deciduous species under seasonal drought stress. These results suggest that evergreen and deciduous woody species adopt conservative and acquisitive water use strategies, respectively (Eamus 1999, Ishida et al. 2010, Poorter et al. 2010, Qi et al. 2021). Drought-deciduous tree species maintain higher water transport via an efficient vascular system and carbon gain in the rainy seasons, and maintain a high P_min_ and drop their leaves to avoid drought damage during seasonal drought (Bartlett et al. 2016); in contrast, on average, evergreen trees can maintain a more negative P_min_ to tolerate seasonal drought, but this comes at the cost of lower xylem hydraulic efficiency throughout the year (Goldstein et al. 1989, Choat et al. 2005, Chen et al. 2009, Ishida et al. 2010, Fu et al. 2012).

We also found significant differences in MOR, MOE, WD, FWT, NDF and ADF values between the evergreen and deciduous groups, indicating a divergence in mechanical strength in the xylem of Chinese savanna woody species with contrasting leaf phenology. In this study, evergreen species developed high-density xylem (higher WD) and thicker walled vessels and fibers (VWT and FWT), as well as investing more support costs in xylem DM (higher NDF and ADF), to enhance xylem biomechanical strength (higher MOR and MOE) compared with the deciduous group. Greater xylem mechanical stability in evergreen species can promote higher resistance to bending and twisting, and can reduce damage by herbivores compared with that experienced by deciduous species (Niklas 1992, Read and Stokes 2006, Onoda et al. 2010).

Our results showed that hydraulic and mechanical traits differed between deciduous and evergreen species in the Chinese savanna, which was driven by the selective pressure of seasonal drought. These results provide insights into conservative and acquisitive life-history strategies for evergreen and deciduous species, respectively. Specifically, deciduous species maintained a higher water transport capacity and promoted fast growth during the rainy season when water availability was abundant, whereas they shed leaves when water was limiting; in contrast, evergreen species exhibited a higher mechanical strength at the cost of a reduction in hydraulic efficiency.

### The trade-offs between hydraulic efficiency and mechanical strength are related to structural and anatomical traits

With or without considering phylogenetic effects, there was a significant and negative correlation between K_s_ and MOR across species or within evergreen and deciduous species, separately. This result revealed a trade-off between hydraulic efficiency and mechanical strength, with or without considering phylogeny, which is consistent with previous studies (Wagner et al. 1998, Fan et al. 2017, Xiao et al. 2021). It has been suggested that this trade-off reflects the conflicting structural requirements for xylem design and fitness in plant species (Baas et al. 2004, Zhang et al. 2013). At the xylem tissue scale, the partitioning of vessels and fibers provides dual functionality of water transport and mechanical support (Lachenbruch and McCulloh 2014, Pratt and Jacobsen 2017, Pratt et al. 2021*a*). Moreover, the traits related to mechanical strength (MOR, MOE, WD, NDF, ADF and FWT) and drought exposure (−P_min_) clustered together on the positive site of PC axis 1, whereas the traits related to water transport efficiency (K_s_, K_L_ and D_h_) clustered together on the negative side of PC axis 1 (Figure 1). Thus, our results imply that the trade-offs between hydraulic efficiency and mechanical strength are related to structural and anatomical traits.

Hydraulic conductivity increases with the fourth power of vessel diameter according to the Hagen-Poiseuille law (Tyree and Zimmermann 2002), and wider and longer vessels would contribute to a greater water flow rate for a given pressure gradient (Choat et al. 2005). A small increase in D_h_ can greatly alter xylem hydraulic efficiency (Tyree and Ewers 1991, Tyree et al. 1994). In the present study, we found that the water transport ability in the xylem (K_s_) was significantly and positively associated with D_h_ across 20 savanna woody species and within the deciduous group (Figure 3). However, K_s_ was affected by MVL in neither Pearson’s correlation nor PICs. This result suggested that xylem hydraulic efficiency was determined by D_h_, rather than by MVL, which was consistent with previous studies (Sperry et al. 2005, Jacobsen et al. 2015). The reason for this result may be that the vessel length was not directly connected to vessel width, and xylem water transport was determined not only by vessel size but also by the inter-vessel pit properties, such as pit area and pit ultra-microstructure (Sperry et al. 2005, Wheeler et al. 2005, Lens et al. 2011, Jacobsen et al. 2015). In this study, we found a close correlation between K_L_ and D_h_ across 20 species and within deciduous group, but no relationship between K_L_ and HV. Xylems with wider D_h_ can have a higher K_s_, and a concurrent increase in K_L_ in Chinese savanna woody species.

The present study also reveals that there is a close correlation between mechanical strength (e.g., MOR and MOE) and fiber content in the xylem (e.g., NDF and ADF), both across species and within evergreen and deciduous species. A previous study has shown that a high fiber content contributes to higher mechanical resistance in leaves (Onoda et al. 2011). To our knowledge, this is the first study to test the associations between fiber chemical composition and xylem mechanical traits. High mechanical strength of the xylem can be achieved by increasing the support cost (e.g., hemicellulose, cellulose and lignin). Our results show that a greater allocation to fiber indeed enhances xylem stiffness and mechanical stability. Moreover, the SMAs results showed marginally different slopes for the regression of mechanical traits and fiber contents between evergreen and deciduous species. Although both MOR and MOE increased with increasing NDF and ADF, there was a higher mechanical strength and stiffness for a given fiber content per volume in the evergreen group than in the deciduous group. Plants may alter the anatomical design of the xylem to enhance mechanical strength, e.g., increasing FWT, altering cellulose microfibril angle and fiber arrangement (Chave et al. 2009, Pratt and Jacobsen 2017). In this study, we found correlations between MOR and FWT, and between MOE and FWT across species; however, these correlations weakened or became insignificant when only considering the evergreen or deciduous group. The significant relationship between MOR and FWT, and between MOE and FWT across species may be due to the divergence of evergreen and deciduous species in mechanical and fiber traits, and was clustered at different positions. Our results suggest that fiber content per volume, rather than fiber anatomical traits, strongly affects xylem mechanical strength.

Our results showed that WD was negatively associated with hydraulic conductivity, but positively associated with mechanical strength and stiffness. Wood density is a comprehensive functional trait that can be used to indicate the conflicting demands for water transport and biomechanics (Santiago et al. 2004, Pratt et al. 2007, Markesteijn et al. 2011, Rosell et al. 2014, Pratt and Jacobsen 2017). It has been suggested that WD is mainly affected by the fiber wall and lumen fractions in the xylem (Ziemińska et al. 2013). With wider vessels in the xylem, there is less space for lignified fiber cells (Wagner et al. 1998). In this study, there was a negative correlation between WD and D_h_, but a positive correlation between WD and FWT, and between WD and VWT across species in both Pearson’s analyses and PICs. A high WD was associated with a thicker fiber wall and higher fiber content, but a lower D_h_, which has been proposed to provide higher mechanical strength against breakage at the cost of hydraulic efficiency (Jacobsen et al. 2005, Plavcová et al. 2019). These structural and chemical constraints govern the trade-offs between xylem hydraulic efficiency and mechanical strength (Pratt and Jacobsen 2017).

## Conclusions

In summary, we found a distinct divergence in hydraulic efficiency and mechanical strength in Chinese savanna species with contrasting leaf phenology, revealing conservative and acquisitive life-history strategies for evergreen and deciduous species, respectively. Our results also showed trade-offs between hydraulic efficiency and mechanical strength across Chinese savanna species, and these trade-offs were modulated by a suite of traits, including WD, vessel and fiber traits and fiber content. Taken together, xylem hydraulic efficiency was mainly driven by D_h_, whereas high levels of xylem mechanical strength were associated with WD, NDF and ADF. The pairwise relationships between traits were phylogenetically independent. The differentiation in stem hydraulics and biomechanics between evergreen and deciduous species suggests divergent life-history strategies, which are closely linked to plant growth and performance. These findings illustrate the hydraulic and biomechanical adaptations of Chinese evergreen and deciduous savanna species to long-term dry-hot environments. It is increasingly critical for future research to explore how hydraulic and mechanical functions affect plant performance in terms of growth, survival and reproduction, and so species fitness. Furthermore, previous studies have suggested some coordination or trade-offs among mechanical support, hydraulic efficiency and safety, storage of water, carbohydrates, nutrients and defensive secondary chemical compounds in wood (Pratt et al. 2007, Chave et al. 2009, Zhang et al. 2013, Plavcová et al. 2019, van der Sande et al. 2019, Pratt et al. 2021*a*). Therefore, further studies are needed to test the potential associations of the efficiency–safety–mechanics–capacitance correlations in xylems to understand the conflicting structural and functional requirements for xylem design and, correspondingly, on plant performance within ecosystems and across ecosystems.

## Authors’ contributions

S.-B. Z. designed the experiment; S.-B. Z., G.-J. W., Y.-Y. Q. and L.-Y.Y. collected the data; S.-B. Z. and Y. S. analyzed the data; and S.-B. Z. led the writing. All authors contributed critically to the drafts and gave final approval for publication.

## Supplementary Material

Supplemental_data_for_online_publication_tpac017
