# Supplementary material for: Trade-offs between xylem hydraulic efficiency and mechanical strength in Chinese evergreen and deciduous savanna species
Source: Tree Physiol. 2022 Feb 14;42(7):1337–49. doi: 10.1093/treephys/tpac017 (PMC9272745; doi:10.1093/treephys/tpac017)
Supplement: Supplemental_data_for_online_publication_tpac017 [file supplemental_data_for_online_publication_tpac017.docx]

Table S1. The results of the Shapiro-Wilk test for 15 functional traits in a savanna ecosystem, Southwest China. Trait abbreviations are shown in Table 2.

| **Functional traits** | ***W*** | ***P*** |
| --- | --- | --- |
| K_s_ | 0.925 | 0.123 |
| K_L_ | 0.874 | **0.014** |
| HV | 0.956 | 0.465 |
| MVL | 0.979 | 0.919 |
| MOR | 0.920 | 0.100 |
| MOE | 0.958 | 0.500 |
| D_h_ | 0.925 | 0.123 |
| VD | 0.895 | **0.033** |
| VWT | 0.937 | 0.209 |
| FWT | 0.930 | 0.157 |
| FLD | 0.919 | 0.096 |
| WD | 0.977 | 0.883 |
| NDF | 0.963 | 0.610 |
| ADF | 0.975 | 0.860 |
| P_min_ | 0.934 | 0.185 |

**Table S2.** Summary of the standardized major axis (SMA) regressions between hydraulic efficiency (K_s_), biomechanical strength (MOR) and functional traits of evergreen species (n=7) and deciduous species (n=13) in a savanna ecosystem, Southwest China. Trait abbreviations are shown in Table 2. Explained variance (*R*^2^), slope, intercept, and 95% confidence intervals of slopes and intercepts across all species and within evergreen and deciduous groups are shown. Significance levels: ^*^, *P* < 0.05; ^**^, *P* < 0.01; ^***^, *P* < 0.001, *ns*, non-significant. The SMA slopes and intercepts between evergreen and deciduous groups were significantly different when the significance was less than 0.05 (*P* < 0.05).

| **Trait 1**  **(*Y*)** | **Trait 2**  **(*X*)** | **Group** | **N** | ***R*^2^** | **Slope** | **Intercept** | **Difference**  **in slope** | **Difference**  **in intercept** |
| --- | --- | --- | --- | --- | --- | --- | --- | --- |
| K_s_ | MOR | All species | 20 | 0.63^***^ | -0.06 [-0.09, -0.05] | 8.28 [6.53, 10.04] | ***P* < 0.001** | *P*  = 0.40 |
|  |  | Deciduous | 13 | 0.71^***^ | -0.10 [-0.15, -0.05] | 11.31[8.50, 14.14] |  |  |
|  |  | Evergreen | 7 | 0.69^*^ | -0.02 [-0.03, -0.01] | 3.28 [1.93, 4.63] |  |  |
| K_s_ | MOE | All species | 20 | 0.80^***^ | -0.0008 [-0.001, -0.0005] | 7.99 [6.26, 9.72] | ***P* < 0.001** | *P* = 0.99 |
|  |  | Deciduous | 13 | 0.51^**^ | -0.001 [-0.002, -0.0007] | 9.82 [6.81, 12.83] |  |  |
|  |  | Evergreen | 7 | 0.74 ^*^ | -0.0003 [-0.0004, -0.0001] | 3.35 [2.06, 4.63] |  |  |
| Log (K_L_) | MOR | All species | 20 | 0.54^***^ | -0.03 [-0.04, -0.02] | 3.76 [2.83, 4.71] | *P* = 0.08 | *P* = 0.84 |
|  |  | Deciduous | 13 | 0.18*ns* | -0.04 [-0.07, -0.02] | 4.63 [2.71, 6.56] |  |  |
|  |  | Evergreen | 7 | 0.53*ns* | -0.02 [-0.04, -0.01] | 2.50 [0.73, 4.27] |  |  |
| Log (K_L_) | MOE | All species | 20 | 0.53^***^ | -0.0004 [-0.0005, -0.0003] | 3.62 [2.71, 4.53] | *P* = 0.13 | *P* = 0.21 |
|  |  | Deciduous | 13 | 0.07 | -0.0005 [-0.0008, -0.0003] | 4.03 [2.34, 5.72] |  |  |
|  |  | Evergreen | 7 | 0.86^**^ | -0.0003 [-0.0004, -0.0001] | 2.58[1.60, 3.56] |  |  |
| K_s_ | D_h_ | All species | 20 | 0.77 ^***^ | 0.13 [0.10, 0.16] | -2.90 [-4.32, -1.48] | *P* = 0.59 | *P* = 0.45 |
|  |  | Deciduous | 13 | 0.62 ^**^ | 0.14 [0.10, 0.21] | -3.81 [-6.91, -0.72] |  |  |
|  |  | Evergreen | 7 | 0.34 *ns* | 0.11 [0.05 0.26] | -2.30 [-5.63, -1.03] |  |  |
| K_s_ | log (VD) | All species | 20 | 0.28 ^*^ | -0.03 [-0.04, -0.02] | 5.73 [4.24, 7.22] | ***P* < 0.01** | *P* = 0.90 |
|  |  | Deciduous | 13 | 0.03 *ns* | -0.05 [-0.08, -0.02] | 7.21 [4.48, 9.94] |  |  |
|  |  | Evergreen | 7 | 0.28 *ns* | -0.01 [-0.03, -0.01] | 2.58 [1.06, 4.09] |  |  |
| **Table S2 continued** | | |  |  |  |  |  | |
| K_s_ | WD | All species | 20 | 0.54 ^***^ | -16.22 [-22.58, -11.66] | 12.28 [9.01, 15.53] | ***P* < 0.01** | *P* = 0.19 |
|  |  | Deciduous | 13 | 0.32^*^ | -19.06 [-32.12, -11. 31] | 13.85 [8.16, 19.53] |  |  |
|  |  | Evergreen | 7 | 0.73 ^**^ | -6.00 [-10.52, -3.41] | 5.28 [2.86, 7.69] |  |  |
| K_s_ | VWT | All species | 20 | 0.28 * | -1.31 [-1.97, -0.87] | 10.57 [7.19, 13.95] | *P* = 0.55 | *P* = 0.13 |
|  |  | Deciduous | 13 | 0.30 ns | -1.15 [-2.07, -0.64] | 9.93 [5.83, 14.04] |  |  |
|  |  | Evergreen | 7 | 0.03 ns | -0.83 [-2.20, -0.31] | 6.87 [-0.44, 12.29] |  |  |
| MOR | FWT | All species | 20 | 0.47 ^***^ | 13.23 [9.30, 18.82] | 4.11 [-27.52, 35.73] | *P* = 0.26 | *P* = 0.11 |
|  |  | Deciduous | 13 | 0.26 *ns* | 15.82 [9.18, 27.24] | -3.40 [-49.44, 41.53] |  |  |
|  |  | Evergreen | 7 | 0.02*ns* | 29.05 [10.96, 76.97] | -12.74 [-433.97, 148.49] |  |  |
| MOR | FLD | All species | 20 | 0.00 *ns* | 10.24 [-6.35, -16.49] | -47.99 [-117.40, 21.41] | *P* = 0.12 | ***P* < 0.01** |
|  |  | Deciduous | 13 | 0.01 *ns* | -5.31 [-9.87, -2.86] | 144.36 [95.96, 192.77] |  |  |
|  |  | Evergreen | 7 | 0.07 *ns* | 12.59 [4.85, 32.70] | -52.52 [-237.44, 132.40] |  |  |
| MOR | WD | All species | 20 | 0.78 ^***^ | 255.10 [202.73, 320.98] | -62.68[-97.92, -27.43] | *P* = 0.05 | *P* = 0.63 |
|  |  | Deciduous | 13 | 0.60 ^**^ | 182.03 [121.22, 273.35] | -24.15 [-65.64, 17.33] |  |  |
|  |  | Evergreen | 7 | 0.82 ^**^ | 325.38 [204.20, 518.47] | -108.62 [-215.55, -1.69] |  |  |
| MOR | NDF | All species | 20 | 0.83 ^***^ | -233.00 [189.72, 286.17] | -31.20 [-36.30, -6.11] | *P* = 0.07 | *P* = 0.98 |
|  |  | Deciduous | 13 | 0.64 ^***^ | 179.38 [121.86, 264.04] | -7.02 [-39.67, 25.63] |  |  |
|  |  | Evergreen | 7 | 0.92 ^***^ | 294.30 [192.44, 450.08] | -68.63 [-149.22, 10.96] |  |  |
| MOR | ADF | All species | 20 | 0.78 ^***^ | 246.86 [195.64, 311.49] | -29.64 [-57.74, -1.53] | ***P* = 0.06** | *P* = 0.74 |
|  |  | Deciduous | 13 | 0.64 ^**^ | 182.05[123.47, 268.43] | -2.46 [-33.51, 28.56] |  |  |
|  |  | Evergreen | 7 | 0.73 ^*^ | -79.04 [-192.02, 22.94] | -79.04 [-192.02, 33.94] |  |  |

**Table S3.** GenBank accessions used to estimate the phylogeny of 20 sampled species.

| **Species** | **Species used** | ***rbc*L** |
| --- | --- | --- |
| *Haldina cordifolia* |  | X83639 |
| *Cipadessa cinerascens* |  | MN126582 |
| *Polyalthia cerasoides* |  | MH069829 |
| *Vitex negundo* |  | MT473785 |
| *Trigonostemon tuberculatum* | *Trigonostemon villosus* | MK876720 |
| *Bauhinia brachycarpa* |  | MF135595 |
| *Lannea coromandelica* |  | KX527147 |
| *Terminalia franchetii* |  | KF753936 |
| *Bridelia stipularis* |  | MW357611 |
| *Strophioblachia fimbricalyx* |  | AY794901 |
| *Terminthia paniculata* |  | ZSB01001 |
| *Woodfordia fruticosa* |  | MK881637 |
| *Campylotropis delavayi* |  | KY702629 |
| *Tarenna depauperata* | *Tarenna sp* | MF435299 |
| *Pistacia weinmanniifolia* |  | KX527077 |
| *Diospyros yunnanensis* |  | KU379486 |
| *Olea europaea* |  | MG255766 |
| *Psidium guajava* |  | KX364403 |
| *Carissa spinarum* |  | AJ419738 |
| *Burretiodendron kydiifolium* | *Burretiodendron esquirolii* | AY328194 |

**Table S4.** Phylogenetic signal, quantified as Blomberg’s *K*, for 15 traits across 20 evergreen and deciduous species in a savanna ecosystem, southwest China. Trait abbreviations were shown in Table 2.

| **Functional traits** | ***K*** | ***P*-values** |
| --- | --- | --- |
| K_s_ | 0.329 | 0.803 |
| K_L_ | 0.347 | 0.781 |
| HV | 0.577 | 0.269 |
| MVL | 0.546 | 0.356 |
| MOR | 0.604 | 0.235 |
| MOE | 0.485 | 0.446 |
| D_h_ | 0.506 | 0.384 |
| VD | 0.842 | 0.302 |
| VWT | 0.300 | 0.878 |
| FWT | 0.453 | 0.617 |
| FLD | 0.611 | 0.209 |
| WD | 0.520 | 0.399 |
| NDF | 0.522 | 0.406 |
| ADF | 0.453 | 0.542 |
| P_min_ | 0.581 | 0.304 |

**Table S5.** The coefficients of Pearson’s correlations (20 species; lower left diagonal) and phylogentically independent contrasts (PICs) (19 contrasts; upper right diagonal) among 15 functional traits. ^*^ 0.01< *P* < 0.05, ^**^ 0.001< *P* < 0.01，^***^*P* < 0.001.

|  | K_s_ | K_L_ | HV | MVL | MOR | MOE | D_h_ | VD | VWT | FWT | FLD | WD | NDF | ADF | -P_min_ |
| --- | --- | --- | --- | --- | --- | --- | --- | --- | --- | --- | --- | --- | --- | --- | --- |
| K_s_ | - | 0.93^***^ | 0.06 | -0.47 | -0.77^***^ | -0.84^***^ | 0.93^***^ | -0.43 | -0.62^*^ | -0.91^***^ | 0.26 | -0.73^**^ | -0.75^***^ | -0.82^***^ | -0.35 |
| K_L_ | 0.85^***^ | 1 | 0.36 | -0.51^*^ | -0.68^**^ | -0.78^***^ | 0.90^***^ | -0.32 | -0.51 | -0.86^***^ | 0.22 | -0.47^*^ | -0.56^*^ | -0.65^**^ | -0.18 |
| HV | 0.16 | 0.62^**^ | 1 | 0.00 | 0.07 | -0.06 | 0.24 | 0.06 | 0.32 | -0.16 | 0.09 | 0.13 | 0.30 | 0.27 | 0.10 |
| MVL | 0.13 | 0.08 | 0.07 | 1 | 0.35 | 0.38 | -0.44 | 0.13 | 0.36 | 0.49 | -0.07 | 0.10 | 0.30 | 0.35 | 0.11 |
| MOR | -0.79^***^ | -0.64^**^ | -0.08 | -0.05 | 1 | 0.93^***^ | -0.72^**^ | 0.61^*^ | 0.57^*^ | 0.76^**^ | -0.03 | 0.88^***^ | 0.92 | 0.90^***^ | 0.62^**^ |
| MOE | -0.78^***^ | -0.61^**^ | -0.08 | -0.01 | 0.91^***^ | 1 | -0.62^*^ | 0.59^*^ | 0.67^**^ | 0.84^***^ | -0.08 | 0.89^***^ | 0.89^***^ | 0.92^***^ | 0.49^*^ |
| D_h_ | 0.88^***^ | 0.83^***^ | 0.36 | 0.13 | -0.75^***^ | -0.76^***^ | 1 | -0.51^*^ | -0.45 | -0.90^***^ | 0.22 | -0.58^*^ | -0.68^**^ | -0.71^**^ | -0.49^*^ |
| VD | -0.54^*^ | -0.43 | -0.18 | -0.03 | 0.51^*^ | 0.52^*^ | -0.68^**^ | 1 | 0.12 | 0.60^*^ | -0.17 | 0.56^*^ | 0.60^*^ | 0.59^*^ | 0.48 |
| VWT | -0.53^*^ | -0.28 | 0.27 | 0.04 | 0.69^**^ | 0.70^***^ | -0.29 | 0.21 | 1 | 0.54^*^ | -0.09 | 0.68^**^ | 0.77^***^ | 0.81^***^ | 0.33 |
| FWT | -0.71^*^ | -0.56^*^ | -0.11 | 0.19 | 0.69^**^ | 0.73^**^ | -0.71^**^ | 0.70^**^ | 0.52^*^ | 1 | -0.14 | 0.74^**^ | 0.74^**^ | 0.75^***^ | 0.48 |
| FLD | 0.16 | 0.23 | 0.2 | 0.18 | 0.00 | -0.02 | 0.09 | -0.10 | 0.02 | 0.11 | 1 | -0.02 | -0.06 | -0.07 | -0.08 |
| WD | -0.75^***^ | -0.56^*^ | 0.24 | 0.29 | 0.92^***^ | 0.88^***^ | -0.70^**^ | 0.56^*^ | 0.72^**^ | 0.71^**^ | -0.21 | 1 | 0.99^***^ | 0.95^***^ | 0.72^**^ |
| NDF | -0.76^***^ | -0.50^*^ | 0.11 | 0.07 | 0.91^***^ | 0.91^***^ | -0.60^*^ | 0.61^*^ | 0.67^**^ | 0.72^**^ | -0.18 | 0.99^***^ | 1 | 0.98^***^ | 0.67^**^ |
| ADF | -0.78^***^ | -0.54^*^ | 0.08 | 0.08 | 0.88^***^ | 0.91^***^ | -0.59^*^ | 0.60^*^ | 0.69^**^ | 0.77^***^ | -0.18 | 0.98^***^ | 0.99^**^ | 1 | 0.55^*^ |
| -P_min_ | -0.58^*^ | -0.47 | -0.14 | 0.10 | 0.69^**^ | 0.63^**^ | -0.62^*^ | 0.59^*^ | 0.33 | 0.70^**^ | 0.01 | 0.71^**^ | 0.71^**^ | 0.68^**^ | 1 |

**
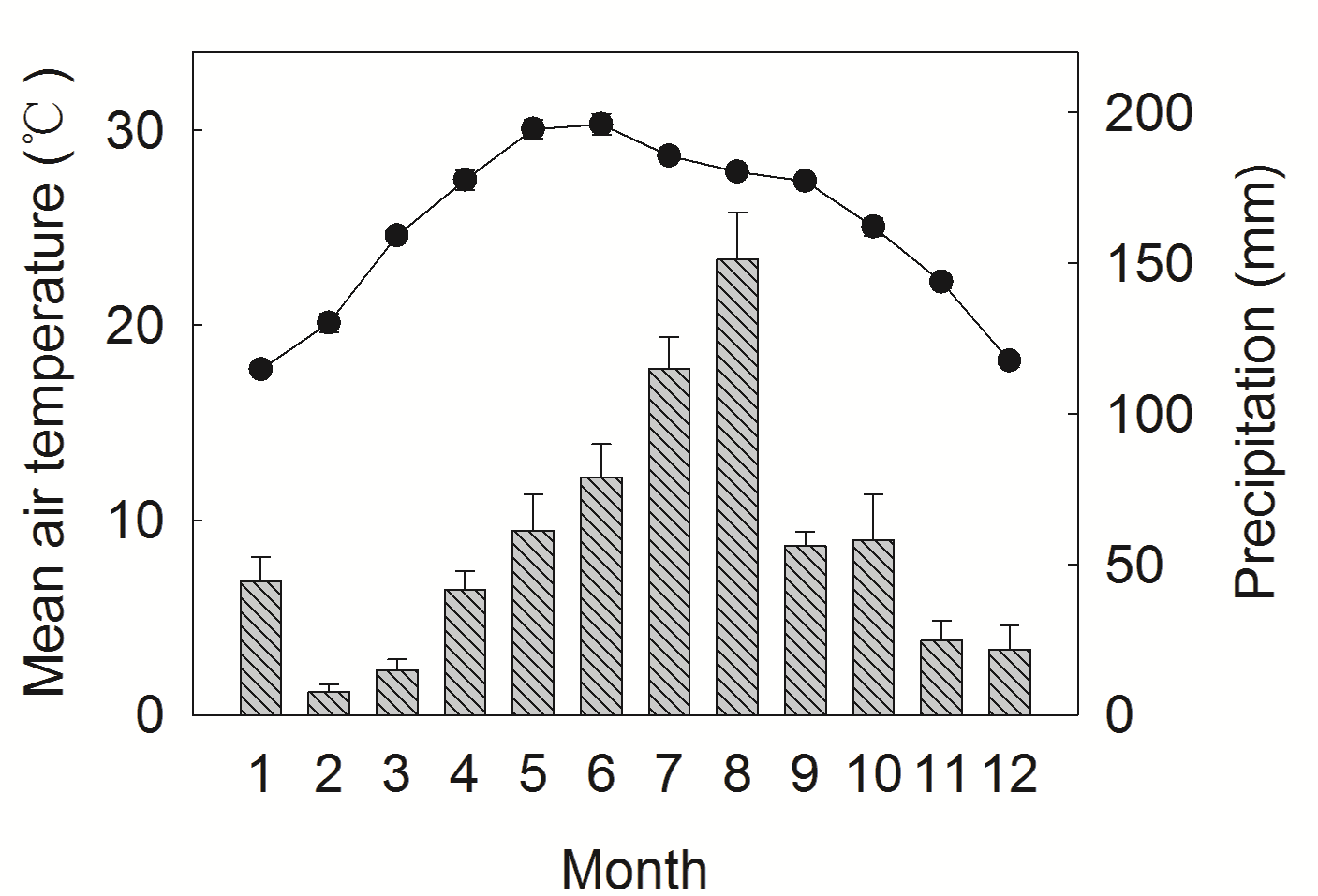
**

**Figure S1.** Mean air temperature (solid circles) and monthly precipitation (vertical bars) for the Yuanjiang savanna ecosystem station for the 2012-2020 period.


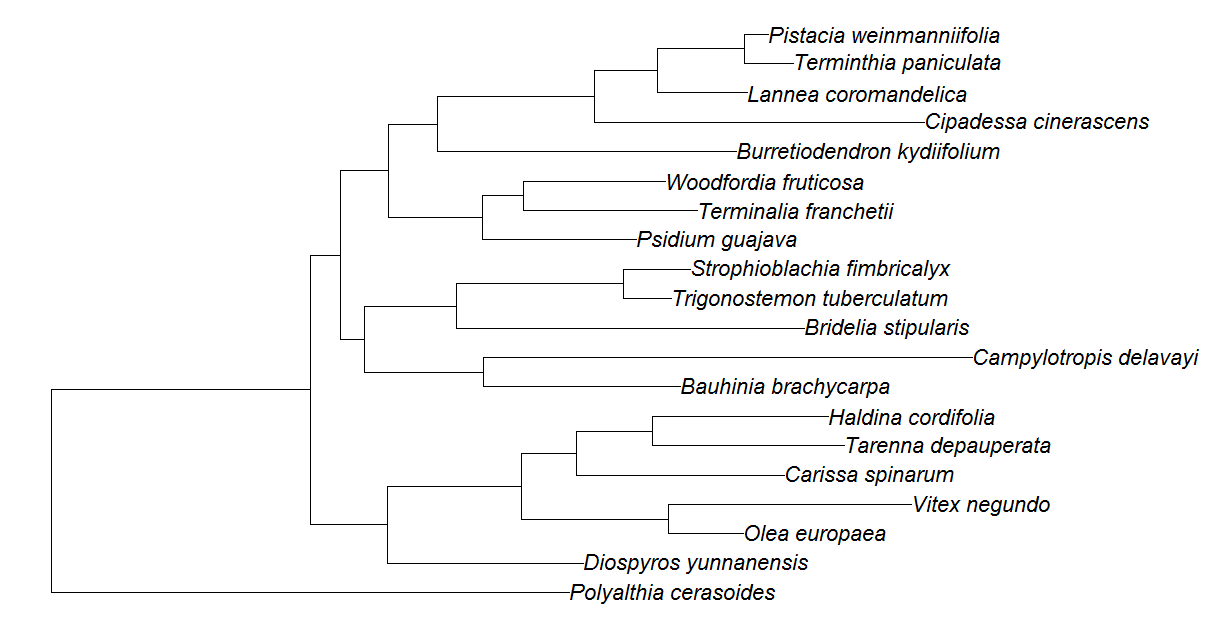


**Figure S2**. Phylogeny tree of twenty studied species from a savanna ecosystem, Southwest China.
